# Blockchain in Healthcare: A Decentralized Platform for Digital Health Passport of COVID-19 Based on Vaccination and Immunity Certificates

**DOI:** 10.3390/healthcare10122453

**Published:** 2022-12-05

**Authors:** Abdul Razzaq, Syed Agha Hassnain Mohsan, Shahbaz Ahmed Khan Ghayyur, Nouf Al-Kahtani, Hend Khalid Alkahtani, Samih M. Mostafa

**Affiliations:** 1 Department of Ocean Technology and Engineering, Ocean College, Zhejiang University, Zhoushan 316021, China; 2 Optical Communications Laboratory, Ocean College, Zhejiang University, Zheda Road 1, Zhoushan 316021, China; 3 Department of Computer Science and Software Engineering, International Islamic University, Islamabad 44000, Pakistan; 4 Department of Health Information Management and Technology College of Public Health, Imam Abdulrahman Bin Faisal University, Dammam 31441, Saudi Arabia; 5 Department of Information Systems, College of Computer and Information Sciences, Princess Nourah bint Abdulrhman University, Riyadh 11671, Saudi Arabia; 6 Department of Computer Science, Faculty of Computers and Information, South Valley University, Qena 83523, Egypt

**Keywords:** COVID-19, blockchain, Smart Contract, Ethereum, distributed storage, IPFS, traceability, vaccination certificates, immunity certificates, digital health passports

## Abstract

COVID-19 has become a very transmissible disease that has had a worldwide impact, resulting in a huge number of infections and fatalities. Testing is critical to the pandemic’s successful response because it helps detect illnesses and so attenuate (isolate/cure) them and now vaccination is a life-safer innovation against the pandemic which helps to make the immunity system stronger and fight against this infection. Patient-sensitive information, on the other hand, is now held in a centralized or third-party storage paradigm, according to COVID-19. One of the most difficult aspects of using a centralized storage strategy is maintaining patient privacy and system transparency. The application of blockchain technology to support health initiatives that can minimize the spread of COVID-19 infections in the context of accessibility of the system and for verification of digital passports. Only by combining blockchain technology with advanced cryptographic algorithms can a secure and privacy-preserving solution to COVID-19 be provided. In this article, we investigate the issue and propose a blockchain-based solution incorporating conscience identity, encryption, and decentralized storage via interplanetary file systems (IPFS). For COVID-19 test takers and vaccination takers, our solution includes digital health passports (DHP) as a certification of test or vaccination. We explain smart contracts constructed and tested with Ethereum to preserve a DHP for test and vaccine takers, allowing for a prompt and trustworthy response from the necessary medical authorities. We use an immutable trustworthy blockchain to minimize medical facility response times, relieve the transmission of incorrect information, and stop the illness from spreading via DHP. We give a detailed explanation of the proposed solution’s system model, development, and assessment in terms of cost and security. Finally, we put the suggested framework to the test by deploying a smart contract prototype on the Ethereum TESTNET network in a Windows environment. The study’s findings revealed that the suggested method is effective and feasible.

## 1. Introduction

The blockchain consists of a list of cryptographically verified data. Databases lack the cryptographic integrity guarantees that are required to function in hostile environments, which is one of the reasons for blockchain’s popularity. It appears that blockchains with privacy and security guarantees are gaining popularity more rapidly [1]. The academic community should work together, now more than ever, to sort the blockchain scams from those which offer valuable contributions [2]. COVID-19 has had a global effect that is unparalleled. Because of its extreme contagiousness, this illness has infected and killed a substantial percentage of the global population. COVID-19 has had a significantly detrimental impact on the global economy as a result of severe mitigating measures; for example, lockdown is a policy used by governments all around the world. COVID-19 symptoms vary in intensity from person to person and the most common symptoms are tiredness, cough, high temperature, and shortness of breath, which are like influenza virus symptoms. However, not everyone who is afflicted with the illness experiences symptoms. Silent carriers, also known as silent spreaders, are those who do not show any signs of the sickness but carry it and pass it on to others [3]. Furthermore, the illness has a fourteen-days duration. Because an infected person might infect people despite suffering no symptoms during this time, thorough and varied testing is required for a successful COVID-19 response.

Blockchain technology [4] has the potential to revolutionize the healthcare business by introducing new models for securely managing and handling healthcare records while also maintaining regulatory compliance [5]. Blockchain technology can be utilized to keep track of public health data; according to these findings, especially during infectious disease outbreaks like COVID-19. During a virus outbreak, a blockchain might be used to report active and recovered patients, allowing local and federal officials to prepare medical facilities and speedy treatments, reducing the epidemic’s spread. However, research findings show that while examining data relevant to the COVID-19 disaster, a significant challenge in data management faced by medical practitioners and researchers was a lack of data integration, authenticity, and point of origin [6]. The bulk of data shared between hospitals, government organizations, and public/private partnerships was inconclusive or difficult to disseminate, according to the Centers for Disease Control (CDC) and Prevention and the World Health Organization (WHO).

Governments develop and implement response strategies based on data linked to illnesses gathered by regional units, as shown during the COVID-19 pandemic [7,8]. Medical findings from hospitals and other specialized institutions are the source of this information. However, the existence of numerous intermediates in this process causes reporting delays, thereby limiting hospital and testing centers’ ability to respond quickly when illnesses are reported [9]. Furthermore, a tiered reporting structure may result in inconsistencies, negatively impacting global response strategies and their capacity to minimize disease.

The purpose of this research is to address the issue of timely and correct reporting of COVID-19 viruses in order to aid disease response efforts. The technology of blockchain has ushered in a new era of application development, one that is heavily reliant on the Bitcoin app’s data structure being implemented correctly [10]. There are numerous real-world applications in a variety of fields, including the IoT [5], e-Government [6], and managing electronic data management [11], which have recently been created. Because of the peer-to-peer network’s self-cryptographic validation structure and the public availability of a distributed ledger of transaction records, these applications employ blockchain technology and smart apps [12,13].

### 1.1. Vaccination Certificates

Some solutions provide test results and, to some extent, information about immunization [14]. After the certificate of immunization testing, the necessity for vaccination-based DHP has become crucial. Immunity testing, on the other hand, is a centralized system which may be less secure than a decentralized system. A completely decentralized public blockchain is unquestionably the better alternative, with each node increasing the network’s scalability, security, and dependability [15,16].

Smart contracts and data from blockchains are both utilized by the peer-to-peer content-based protocol known as the Interplanetary File System (IPFS). The blockchain’s smart contracts provide several benefits, including efficiency, security, trust, speed, accuracy, and transparency [17]. Decentralizing the certificates and lowering the burden on the data server are made possible by IPFSs higher bandwidth. The certificate of cipher text that has been encrypted is uploaded to IPFS storage. IPFS moves data over a hash string route. It is used to store encrypted data as well as other information. The routes function in a similar way to the web’s standard universal resource locator. As a consequence, all medical data may be accessed at any time by utilizing their hash.

We offer a cutting-edge blockchain-based method for establishing trust and preventing fraud. Our system makes use of smart contracts with the Ethereum platform to run a task and produce events that alert participants to medical information, test changes, and needs. Furthermore, using DHP and immunity certificates stored on the blockchain, our approach aids in the prevention of the COVID-19 virus. Because the data on the blockchain is immutable, all data is gathered from reliable sources with connections to higher levels of government, such as the Ministry of Foreign Affairs (MoFA), the Ministry of Health (MoH), and COVID-19 checking centers. We have also included self-sovereign identification, encryption methods, and decentralized storage systems in our solution.

### 1.2. Contributions of the Study

**Figure 1:**
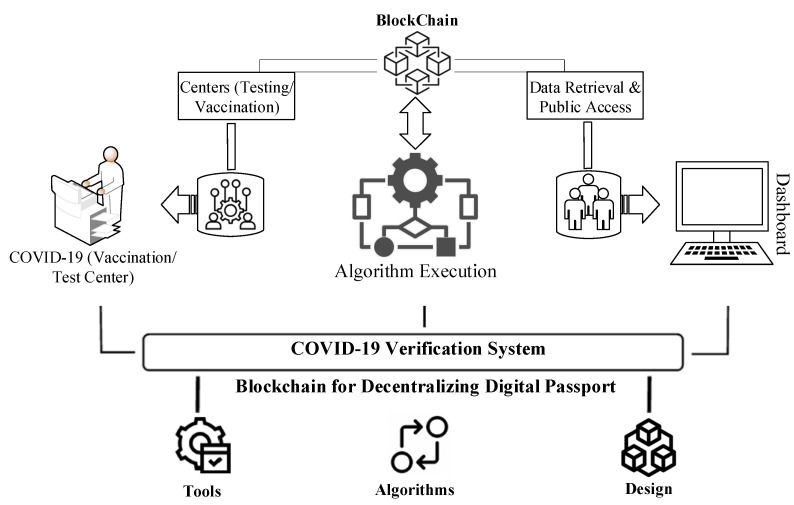
A high-level overview of the suggested Model.

The proposed system of our developed platform has the structure shown in Figure 1, and it provides guidance for maintaining the system of each module of abstraction, i.e., steps in modules allow different operational aspects to be structured as different layers of the system, and the modulation of algorithmic requirements. The following are the primary contributions we have made to this article:

1. To give a high-level overview of the proposed Digital Passport system’s structure and show how the system’s various components interact.
2. We have proposed a blockchain-based approach that allows COVID-19 test takers to be tracked and traced. The proposed method makes use of the distributed blockchain ledger’s immutable events and decentralized identity system.
3. We have used encryption techniques to save the medical report, identification, and travel information of patients and test-takers utilizing our blockchain-based technology and the Inter-Planetary File System (IPFS). In addition, we conduct a security and economic study of our solution in order to establish its viability and dependability.
4. We have used a framework to see if the notion is feasible. In order to do this, we have built a patient-centric control system prototype on the Ethereum test network. The associated source code has been made available on the internet.

We have used test cases to validate functionality and have evaluated the proposed framework’s capabilities: function execution time, accessing time, execution cost, and average gas consumption.

### 1.3. Paper Organization

The remainder of the paper is laid out as follows: Section 2 discusses the background and literature point of view; Section 3 presents the research methodology and the proposed blockchain-based solution’s design details including the smart contracts with execution code; Section 4 explains the implementation of algorithms and the use of technologies. The suggested system is evaluated in Section 5, which is followed by a section that shows the practicality of our approach through a detailed cost and security study. The paper comes to a close with Section 6.

## 2. Legacy Evolutions: Existing View and Emerging Technical Challenges

The reality of sharing health information is slightly more realistic and uncomfortable because we do not live in the future. Imagining health departments in the future presents a difficulty that goes beyond what transferring other patient certificates imply.

Certificates of immunity are meant to demonstrate that a person has acquired the necessary antibodies to protect themselves from COVID-19 and, as a result, is not a risk to others. We believe this was accomplished either by a previous COVID-19 infection or through immunization (where available). We recognize the importance of this problem. However, because our focus is on the technology side of the issue, we have decided to leave the medical aspect of the challenge out of this work [18]. As a result of their immunity to the illness, individuals can be free of physical and social limitations [19]. The health facility can report the results of an antibody test, as well as the duration of the patient’s immunity.

Among scientists and academicians, the immunity period remains a controversial and inconclusive subject. This study, on the other hand, is meant to accommodate scientific advancement and is adaptable to changes based on the findings. There were no published studies between April and July 2020 that demonstrated a safety from a secondary SARS-CoV-2 infection in individuals who had antibodies from the original illness [20]. However, a few recently published studies have found significant evidence of clinical immunity that protects against SARS-CoV-2 re-infection [21,22]. The immunity time frame is still under investigation, although it is thought to be applicable in the short term. To solve this, we have included the moment when an announcement regarding immunity certificates is made in our design. A centralized database has a significant chance of a breach, which could compromise data security. The Equifax data hack, for example, impacted about 140 million people [23].

Ting et al. [24] investigated how a variety of cutting-edge technologies can aid in the prevention of COVID-19 transmission. Furthermore, these technologies can contribute to the development of a screening tool that would aid in disease diagnosis and tracking.

Torky and Hassanien [25], on the other hand, suggested blockchain might be used to spontaneously distinguish infected patients and assess the COVID-19 virus consequence on society. The authors employed blockchain’s decentralized feature to save COVID-19 individuals’ personal information and medical records [26,27]. Other technologies, including an infection verification subsystem, are used to detect infected patients in a system of widespread surveillance. In a related manner, Nguyen et al. [28] presented a method for forecasting the COVID-19 virus and recommended that a huge volume of medical data with a complicated pattern be processed utilizing AI and blockchain. A blockchain-based solution to source tracing and the healthcare supply chain is outlined in detail in the study. However, no technical specifics about the implementation are disclosed.

Bansal et al. [29] additionally demonstrated by utilizing blockchain how to produce immunity certificates. Experts also suggested that an unchangeable blockchain approach should be used to limit the spread of false information and reports. The suggested technique also addresses the privacy and confidentiality concerns of test-takers. The authors, on the other hand, have left out a design plan and a mechanism for completing the proposal’s goals. Resiere et al. [30] offered a solution based on a blockchain for modernizing the system of healthcare. As a result, the experts suggested that in order to combat the spread of COVID-19, blockchain technology be used to enhance medical collaboration and collaborative scientific research. Finally, Kumar et al. [31] developed a strategy for increasing a deep learning network that recognizes COVID-19 patients using different available techniques like CT slices VGG19. AlexNet, ResNet, and MobileNet were used to compare the authors’ methods, as well as other deep learning model blockchains used in their study as a method of exchanging data while protecting anonymity.

A number of approaches in order to make use of sharp-edge technology such as blockchain and the IoT to achieve COVID-19 reaction have been investigated. With the exception of [29], where the authors reveal the implementation details of their deep learning model, prior attempts have lacked technical specifications. Furthermore, none of the options have demonstrated how to use the technology of blockchain to prevent the spread of COVID-19. The bulk of publications have either mentioned blockchain as a viable solution to regulate the spread of ambiguous knowledge or have recommended that it is used in conjunction with other technologies to form a framework [32]. A blockchain-based system was not envisioned or implemented in any of the investigations that would have allowed COVID-19 test-takers or vaccination takers to be traced using associated and authenticated immunity certificates and digital health passports.

Building resilience across the value chain is the industrial industry's biggest problem [33]. Due to the unavoidable disruption brought on by the COVID-19, the scope of agile initiatives has become more significant. When it comes to managing and creating preventive measures, blockchain technology is extremely important. This study [34] has discussed the essential characteristics of blockchain technology and how it may be used to execute a wide range of use cases, including contact tracking, efficient data gathering, safe data storage, and efficient data analysis. Blockchain has the ability to significantly improve COVID-19 data management as compared to centralized database systems for data handling.

In [35], the authors suggest a medical data-sharing network based on blockchain technology. A framework that allows for easy modification tracking will aid in the administration of the patient’s history. A blockchain network will allow several individuals to communicate with the COVID-19 platform. As a result, without the participation of a third party, several sorts of patient certificates can be kept on a network. The data can be stored via IPFS, a decentralized storage system. Smart contracts can be used on Ethereum to make interactions between different entities easier, such as patients, healthcare organizations of governments.

The advantages and disadvantages of a decentralized and centralized identity management system are summarized in Table 1. We have utilized a blockchain-based technique to create digital health passports based on vaccination and immunity certifications. The storage is spread in a decentralized identity management system, and the user has access to DHP. Because it provides security and transparency, the DHP based on immunity and immunization may be validated and uploaded to decentralized storage. As a result, the users’ autonomy is respected and realized. Self-Sovereign Identity (SSI) allows users to control their personally identifiable information (PII), medical records, and immunity certificates. Access rights to credentials, including PII, should be available to SSI holders. Instead of relying on a third party or a monolithic SSI management system, test-takers, patients, and on-chain participants will use a decentralized system. In addition to managing and updating their credentials, they can choose what should be kept private. The security of data in a centralized database is at high risk of being compromised by a breach. With a decentralized SSI, there is no need to rely on centralized databases to prevent data breaches and identity theft.

## 3. Research Methodology and Motivation Consequence

**Figure 2:**
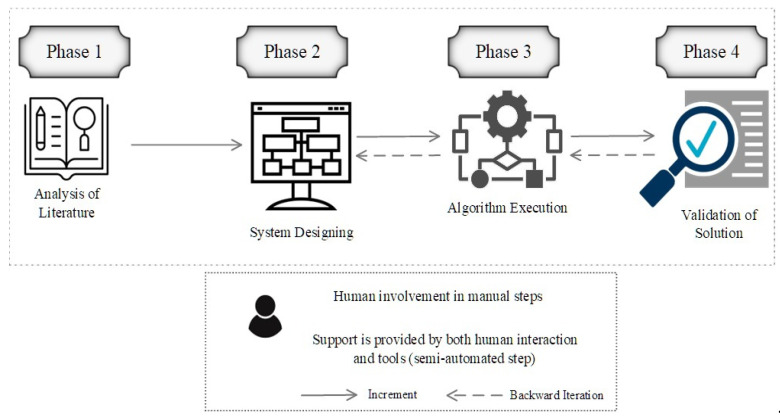
Research Methodology Overview.

We will now go through the study methodology, as well as the design details for the recommended solution (see Figure 2). The design of the proposed blockchain system is thoroughly explained in this section. Our system will use immutable records, trustworthy events, and Ethereum smart contracts. Our plan for tracking and tracing medical test results and travel authorizations for patients, employers, governmental structures, social and intellectual institutions, and transportation networks will all be liberated from the burden of creating an identity for their respective entities. Additionally, it will help to limit and reduce the COVID-19 virus.

**Figure 3:**
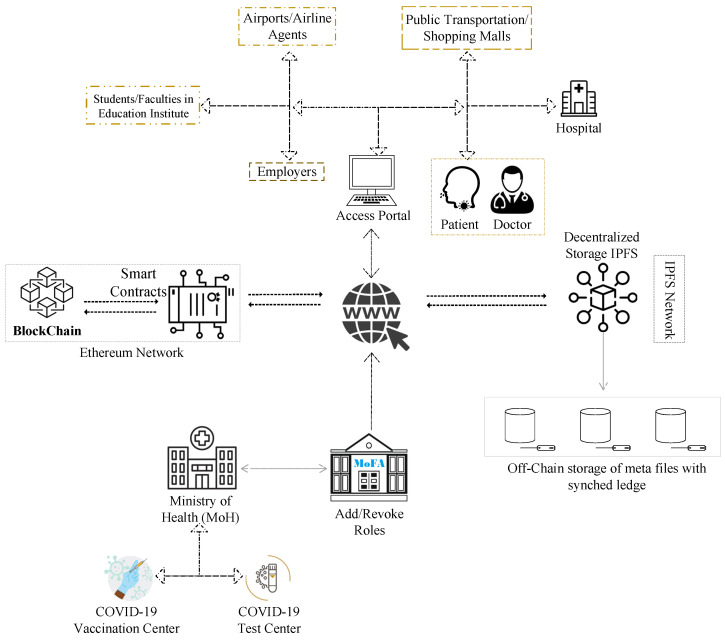
Overview of the proposed solution.

Figure 3 shows a high-level overview of the proposed system. The suggested system’s model provides an overview of the tools and encryption techniques that are employed. The model visualizes communication between all modules and stakeholder groups. The system’s design directs the developer in the implementation of algorithms and the rest of the system.

The suggested blockchain-based solution’s system diagram is shown in Figure 1. It shows on-chain participants, a variety of smart contracts, clients of blockchain, and concerned stakeholders. In our suggested approach, we have employed four different forms of smart contracts: Smart contracts include the MoH, the MoFA, the COVID-19 Testing Center, and the Patient. The smart contracts, as well as other system sub-components, are described in depth below.

DHPs are an important form of verification that can aid in the prevention of disease transmission. This goal will be addressed via the patient smart contract, which is an irreversible certificate that the MoH and the MoFH have both verified for usage. The IPFS hash of a person’s vaccination and immunization data, as well as medical and travel history, is stored in the patient smart contract. Dissemination is the responsibility of the owner of the personally identifiable information used in this structure.

Because immunity to the virus is a good and encouraging development, the statement can be made as a public notification. If the test-taker prefers, there is another method that simply uses IPFS hashes to post changes and test results. The COVID-19 Testing Centre smart contract, according to our design, can perform activities that warn patients and test-takers about medical changes. These updates could contain information concerning quarantine or specifics about medical checks they have undertaken. As a result, the testing center and vaccination center can update the status of immunity testing or vaccination detail, ensuring that it is permanent. On-chain, IPFS hashes will be used to provide private information or medical test results.

### Data Confidential

IPFS is a decentralized storage system for off-chain certificates. The cost of storing papers relevant to COVID-19 testing, identification, and travel on-chain would be prohibitive. As a result, data must be stored in a decentralized and secure manner. IPFS storage is public and widely distributed and as a result, IPFS data should be encrypted, and decrypted data must only be accessible to authorized parties. As a result, in the system design model of the proposed platform, a data handler encrypts the certificate as a DHP with a secret key and uploads it to the IPFS server. Furthermore, the proposed model gives access to several users to view the data while ensuring secrecy [36]. Hospitals, scanning/testing centers, airline agents, airport authorities, and employers are among the organizations that fall within this category. As a result, a method should be implemented that permits material to be shared with the data owner’s consent. The technology should also allow clear content to be accessed by just the authorized recipient.

**Figure 4:**
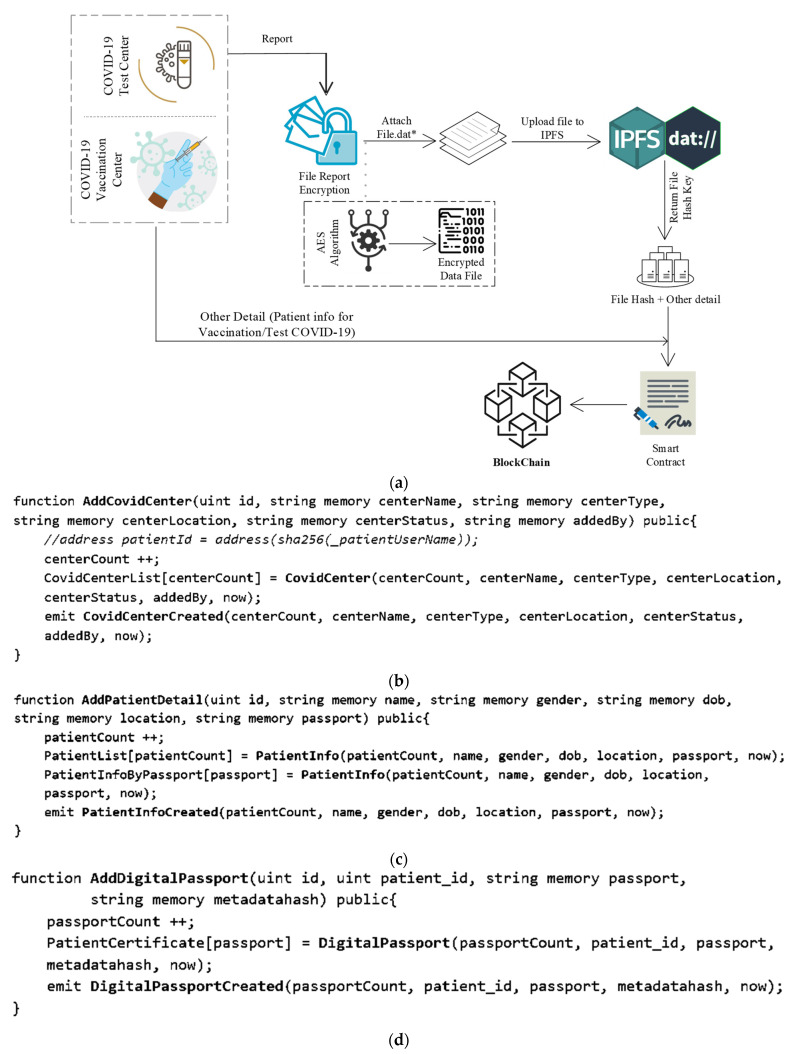
(**a**) Data storing process. (**b**) Smart contract function for adding centers. (**c**) Smart contract function for adding detail of patient. (**d**) smart contract function for creating digital passport.

Figure 4 depicts the process for archiving DHP and test results. A test report is a textual document that a lab assistant instantly deposits into the blockchain via a smart contract. DHP is stored on IPFS in the lab’s health centers section. DHP uploading involves health facilities uploading accessible DHP data to IPFS and receiving a hash key, which is then stored in the blockchain along with other necessary data. Both execution processes are distinct; a test report might be anything, such as a blood test report or a lipid test report, for example. Nevertheless, DHP is a separate component that is recorded in IPFS and then mapped with other needed details to be put in the blockchain.

The generation of metadata for the original file is the first step in the digital data exchange process. Information such as the file’s name, type, description, and size will be included in the DHP metadata. When the metadata is completed, it is combined with the data file and published to IPFS. The following is an example of a file upload to IPFS:

We provide the needed parameters to put the data into the blockchain using a smart contract using the “AddCovidCenter” method described before. This function is written in the Solidity language, which is a smart contract language (see Figure 4b). For searching the data on the portal, we map three distinct mapping sets. The first category is used to obtain a list of all health centers, the second mapping is used to get data by appointment id, and the third mapping is used to access the health centers with an auto-generated id.

Using the “AddPatientDetail” function in a smart contract, we provide the required parameters to save the data into the blockchain (see Figure 4c). We have established three categories to help people discover information on the site. The first category is used to obtain a list of all blood tests, the second mapping is used to obtain data by default produced id, and the passport number is used to gain access to patient data. The “PatientInfoCreated” events is then executed.

The data is saved in the blockchain using a smart contract, and the required parameters are provided using the “AddDigitalPassport” function (see Figure 4d). The mapping is used to retrieve the list of all patients’ certificates using passport numbers. The “DigitalPassportCreated” event is then generated.

**Figure 5:**
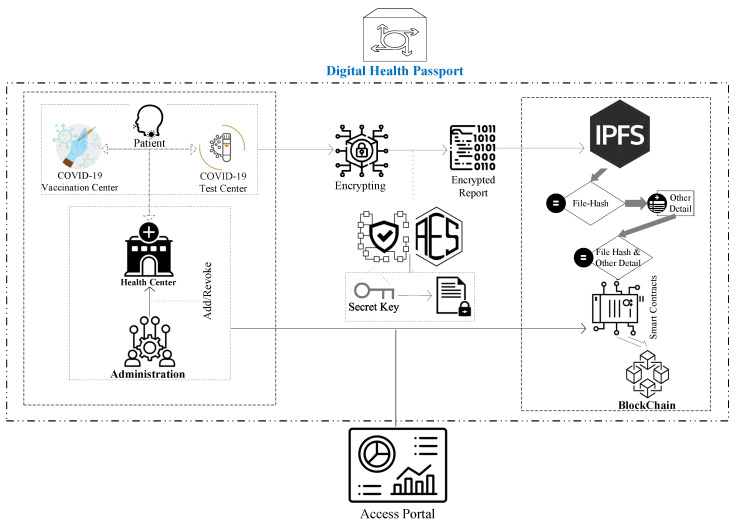
An overview of the detailed solution.

In Figure 5, there are two sorts of medical data uploading categories in DApp. First is the COVID-19 test reporting of patients. The health center stores the record in the blockchain against the patient’s passport number and then the DHP certificate is uploaded to IPFS using the DApp system and provides the rest of the required detail against the patient’s passport number, which is stored in the blockchain.

The dashboard provides the data to both the user-health center and also to a patient; that is, the publicly available data. It is an open-access platform that allows users to view and access DHP certificates that can be downloaded. The DHP certificate may be accessed via a web portal. The stakeholder is able to access the data against the patient’s passport number. The digital passport is secured by using the AES algorithm. Only the patient can convert the downloaded cipher file to a digital passport by passing a secret key; or the allowed healthcare centers can access the patient’s digital passport in a readable form.

## 4. Implementation of Algorithms and Technology for a Solution

In this section, we provide the details of the tools and technology to implement the solution. The suggested system is an Ethereum blockchain private network. Ethereum is a distributed open-source platform that makes good use of Solidity, a programming language that allows smart contracts to be written (scripting language). As a web server, Node.js 15.3.0 has been utilized, together with Truffle 5.3.0, Ganache 2.5.4, and IPFS v.33.1.1. For the DApps networking, we have used 802.11nWiFi.

Details on how to put it in place are provided in this section. A private Ethereum blockchain network is proposed as the system. Ethereum is a decentralized open-source platform that makes extensive use of the Solidity programming language, a programming language that facilitates the creation of smart contracts (scripting language). Node.js 15.3.0 has been used as the web server, together with Truffle 5.3.0, Ganache 2.5.4, and IPFS v.33.1.1. We have used 802.11nWiFi for the DApps networking.

### 4.1. Visual Studio Code

Microsoft’s Visual Studio Code (VSC) is a cross-platform code editor that runs on a variety of platforms. Microsoft’s VSC source-code editor is dual-licensed for Windows, Linux, and macOS. Tools for debugging, syntax highlighting, intelligent code completion, integrated Git control, and code rewriting are all accessible [37].

### 4.2. Ganache

Ganache is a blockchain-based emulator that can run several tests and commands. Ganache is an Ethereum private blockchain that can be used to perform tests, deploy contracts, and build apps. It examines the system’s status and so regulates the blockchain’s operation. It used to be known as Test RPC, but it was renamed Ganache later [38].

### 4.3. Metamask:

Metamask is a web browser add-on that connects to a dispersed network. It executes Ethereum decentralized apps in the browser instead of running the complete Ethereum node. Users may use a browser to log into their Ethereum wallet [39].

### 4.4. Contract creation:

Only a lab assistant, doctor, or patient may create and perform this function to upload, store, and retrieve medical data.

Algorithm 1 is a basic authentication process where patients and other centers are verified and get access to the portal for performing the operation of COVID-19.
**Algorithm 1** Contract Execution1: Input:  L*ζ*(*parameters*)2: Output: R*esult*3:    **procedure** Smart_Contract4:     **if** msg.sender is not Valid **then**5:      throw;6:  **end if**7:     Function Execution ( L*ζparameters*) 8: **end procedure**

This part of Algorithm 2 shows and describes the uploading feature for digital health passports. The method is used to upload data to IPFS and save the hash of the file in a smart contract with a mapping of extra attributes. The file hash of the provided data is connected to a number of factors (User ID, Description, Date, e.g.,).

1. Input(s): With the algorithm’s input, the parameters are mapped to a file hash key.
2. Processing: The hash key is returned after reading the health certificate file and converting it to a buffer package, which is subsequently uploaded to IPFS as a medical data file. The hash key of the provided data is connected to additional parameters. The following information is put into a smart contract and saved on the blockchain: User ID, Appointment ID, Description, and Date.
3. Output: The output is the mapped data, which is kept in the blockchain.

**Algorithm 2** Create Patient Record1: Input:     _(L*(parameters)*)_, T_Type  List of Parameters (Patient Report), Test Type 2: Output: Result            Returning Result3:    **procedure** SavingPatientData4:     **if** T_Type == Vaccination T_Type == COVID-19 Test **then**5:       **if** T_Type == COVID-19 Test **then**6:    R*esult* ← Test(*L*_(*parameters*)_) 7:       **else**8:         R*esult* ← Vaccination(L_(*parameters*)_) 9:       **end if**10:     **end if**11:     Ledger ← Save(R*esult*)       Store to blockchain ledger 12: **end procedure**

This section demonstrates and describes the feature of storing medical records in a blockchain ledger in Algorithm 3. Medical data, such as vaccination certificates and immunity certificates such as digital passports are stored on the blockchain ledger utilizing a smart contract with a mapping of certain extra attributes. Many parameters are stored in the blockchain, including lipid test parameters (Patient User Id, Passport Number, and File Hash of certificate)

1. Input(s): Using the algorithm’s input, the parameters are mapped to the user id and user appointment id.
2. Processing: The medical data is subsequently submitted as a medical data report to the blockchain ledger. For preserving data in the blockchain, additional factors such as user passport, Patient IDs and passport numbers are mapped and saved in a smart contract on the blockchain.
3. Output: The mapped data is stored in the blockchain ledger as the output.

**Algorithm 3** Creating Digital Passport Certificate1: Input: Patient_id, Passport_id, Certificate2: Output:     R*esult*                Returning Result3:    **procedure** DigitalPassport4:      **if** User is authenticated **then**5:          File_Stream ←File_Conversion(Certificate)6:          File_Buffer ← Convert_To_Buffer (File_Stream)7:          Cipher_Data ← AES_Algorithm(Secret_Key, File_Buffer) 8:          File_Hash ← IPFS_ADD(Cipher_Data)9:             R*esult* ← Add_To_Blockchain_Ledger(Patient id, Passport id, Certificate) 10:     **end if**11: **end procedure**

Algorithm 4 verifies data accessing capabilities, which are shown in this section. The algorithm is used to extract information from the blockchain and make it publicly accessible. The user can access blockchain data using the settings they have selected. Data may be accessed in a variety of ways. For example, a user can get data based on their patient id and passport number mapping. Stakeholders can obtain a copy of the DHP certificate by entering the user’s passport number.

1. Input(s): The algorithm’s input is used to map the parameters for accessing the data.
2. Processing: The data from blockchain might be accessible in a variety of ways, such as by a patient id mapped to a passport number.
3. Output: The end result is mapped data that is available to the public.

**Algorithm 4** Interface Layer1: Input: Passport_Number 2:    Output: R*esult*3:       **procedure** Access_Digital_Passport4:       **if** Passport_Number Exists **then**5:         File_Hash ← Get Patient_Record(Passport Number) 6:         Cipher_Data <<- *Get_*IPFS(File_Hash)7:         *DECRYPT ED_Data* ← AES_Algorithm(Cipher_Data) 8:         *Result ←* Download Certificate(*DECRYPTED_Data*)9:       **end if**10:       UpdateDashboard(Result)11: **end procedure**

### 4.5. Tools and Technologies for Algorithmic Implementation

**Figure 6:**
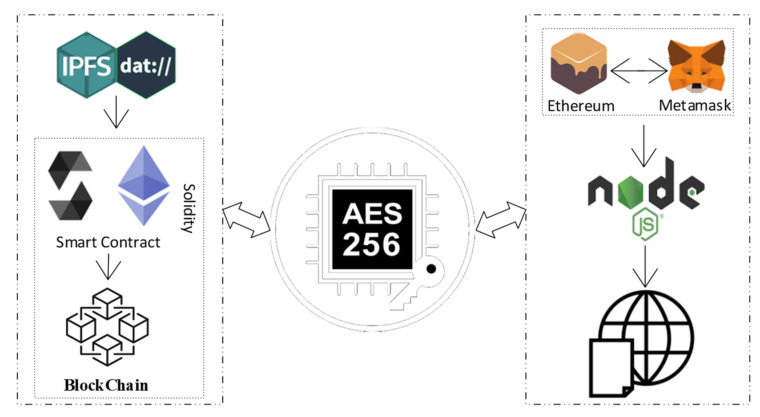
A description of the technologies and tools used for system implementation.

The complementary roles of pertinent tools and technologies in the suggested remedy are summarized in this section. The goal of this discussion is to provide the reader with a better understanding of technology. Technologies and tools are layered, as seen in Figure 6. The data, a DHP certificate file, is uploaded in encrypted form to the IPFS platform and returned as a hash key and stored in the blockchain ledger. A server-side application is made using the NodeJS platform, which provides a variety of tools. The NodeJS application was launched using Visual Studio Code (VSC). We quickly built a personal Ethereum blockchain using the Ganache Truffle Suite package that can be used to perform tests, send instructions, and keep track of states while keeping control over the chain’s operation.

## 5. Results and Evaluation

The outcomes of the proposed approach are shown in this section. The assessment environment comes first, followed by a smart contract functionality evaluation based on fuel use. After that, we have evaluated and quantified data uploading and storage to the blockchain, query response (i.e., performance), and algorithmic execution using criteria (i.e., efficiency). Threats to the research’s validity have also been mentioned, as are any restrictions that must be addressed.

### 5.1. Evaluation Environment

The solution has been tested and its progress through several execution phases and outcomes have been tracked using a set of hardware and software resources called the evaluation environment. On the hardware side, evaluation trials on the Windows Platform employ submissions of lab test results and health certificates to IPFS (core i7 with 16 GB of runtime memory). The automated system testing method known as execution evaluation, or evaluation scripts, is used in the software industry. Similar scripts were written in NodeJS and run in Visual Studio Code using the ReactJS programming language. The review procedure also made use of several already-existing libraries, such as react, web3, ipfs.http. For example, while uploading medical data photos to IPFS and storing them on the blockchain, as well as when retrieving data from the blockchain, a JavaScript performance library script was used to track CPU usage. The Ganache suit was used to establish a local Ethereum blockchain environment, and the Metamask extension was used to connect the distributed web in the browser. The Metamask plugin linked local Ethereum accounts to the Ganache suit, which then performed system operations using the gas transaction cost.

### 5.2. Fuel Consumption and Data Uploading

For the Ethereum smart contract to work, the fuel must be paid for. As a result, the original data transfer’s fuel consumption was assessed and compared to the planned data upload’s fuel usage. Gwei is the smallest unit of Ether, the Ethereum cryptocurrency, and it was used to measure fuel use. Gwei is made up of 10^9^ wei.

In our recommended approach, we stated the cost of contract migration execution (see Table 2). The price is stated in Ether, and the amount of gas used is specified. The amount of gas used multiplied by the price of gas equals ether. The gas represents the system’s ongoing computational costs. The network [26] adjusts the gas price to account for changes in the value of Ether.

Table 2 shows the amount of gas used to execute smart contracts. In the implemented prototype of our system, we set a gas limitation as the default. The Contract was generated only once, for a cost of 0. 03,574,426 Ether at a total gas consumption of 1,787,213. Contract formation at a low cost of 0.0054726 (Ether) and gas usage of just 27,363 were required for the migration. If the amount of input data is kept to a minimum, overall costs can be lowered even further. However, these costs were still less than those associated with employing a centralized system to manage a database like MedChain or renting storage space from a third party [27].

**Figure 7:**
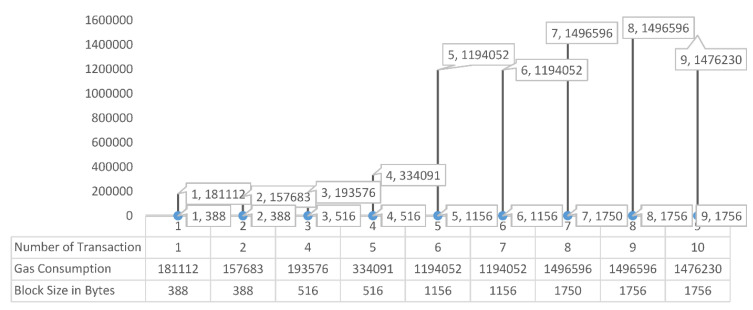
Utilized gas depends on the block size and the number of transactions.

The third test item was the time it took for users to upload and save data to IPFS and the blockchain ledger. Data uploading and accessing time refers to the time spent uploading medical data, retrieving accessible data, and evaluating data. Figure 7 depicts the results of a set of tests with typical data sizes. When uploading 1750+ bytes of data, the average fuel consumption was about 1,476,230, and when storing 380+ bytes of data, the average fuel consumption was around 1,57,683 Gas. This shows that as the data expands in size, so does the amount of fuel consumption. When the medical data was transferred to IPFS following the proposed approach, there was no noticeable difference in fuel usage, despite the fact that the data volume increased.

**Figure 8:**
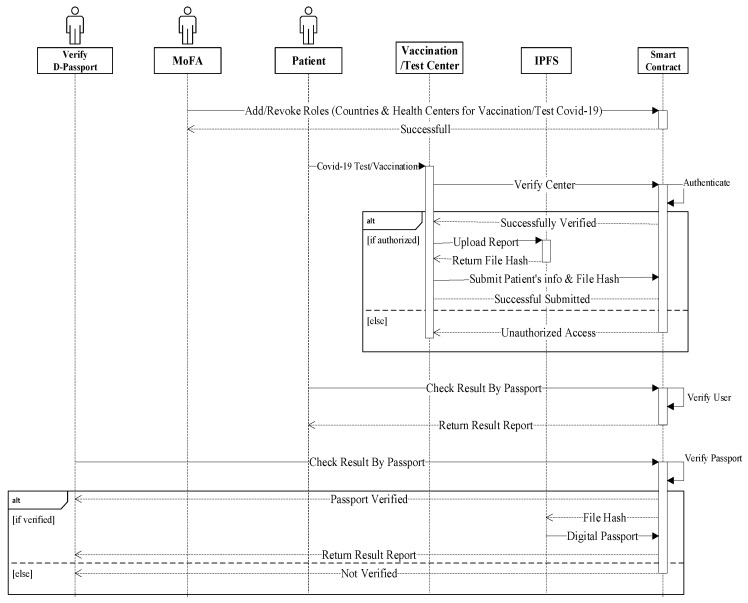
Sequence process for digital passport.

Figure 8 depicts the sequence diagram for all network elements and their interactions. Figure 8 depicts the system’s execution flow. Multiple entities are present (MoFA, MoH, Health Centers, IPFS, Smart Contract). The data from the blockchain ledger was accessed via a web interface by health centers and patient entities. The health center entity was used to submit COVID-19 data (test takers and vaccine takers) to IPFS and get a file hash that was mapped with other needed information parameters and saved in the blockchain.

Using a function named AddCountry, the MoFA can add a nation to its list of associated countries. This function generates an event that informs all listeners of the affiliation of a new country’s MoH. The Ethereum address of the affiliated MoH department is utilized as both an input and output to the function. Dynamic roles are at the heart of the system. The role can be customized to meet the needs of the organization. Roles can be issued to new authorities in order to grant system access, or they can be withdrawn in part or entirely.

**Figure 9:**
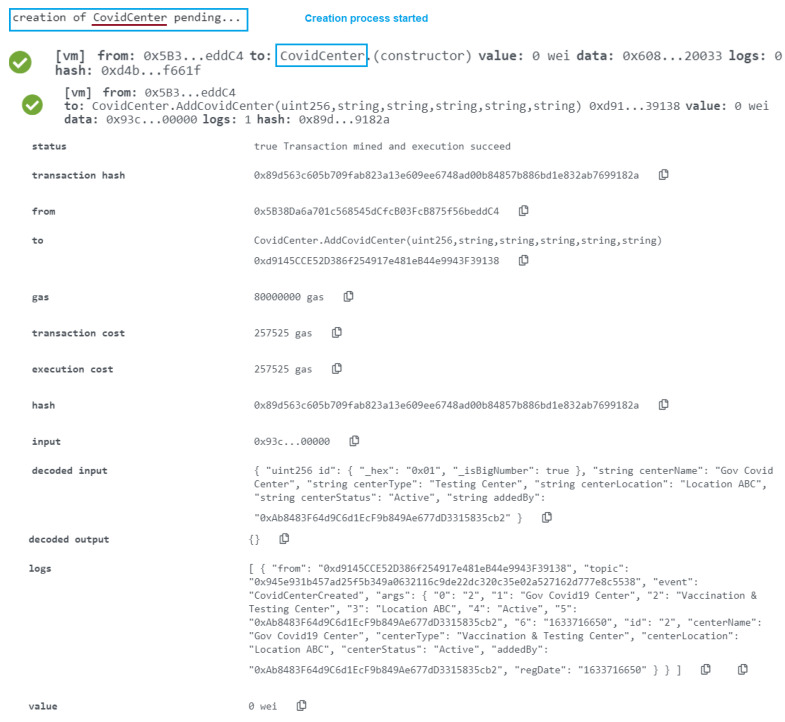
Add COVID-19 Centers in the blockchain ledger.

The approved entity that can use the AddCovidCenter function is specified in the MoH smart contract. The new decision’s effective date, as well as the Ethereum address of the MoH-affiliated testing and immunization facility, are included. This is made known to all interested listeners via an event. The task was completed successfully, as shown in Figure 9, and the event contains the time in uint256.

**Figure 10:**
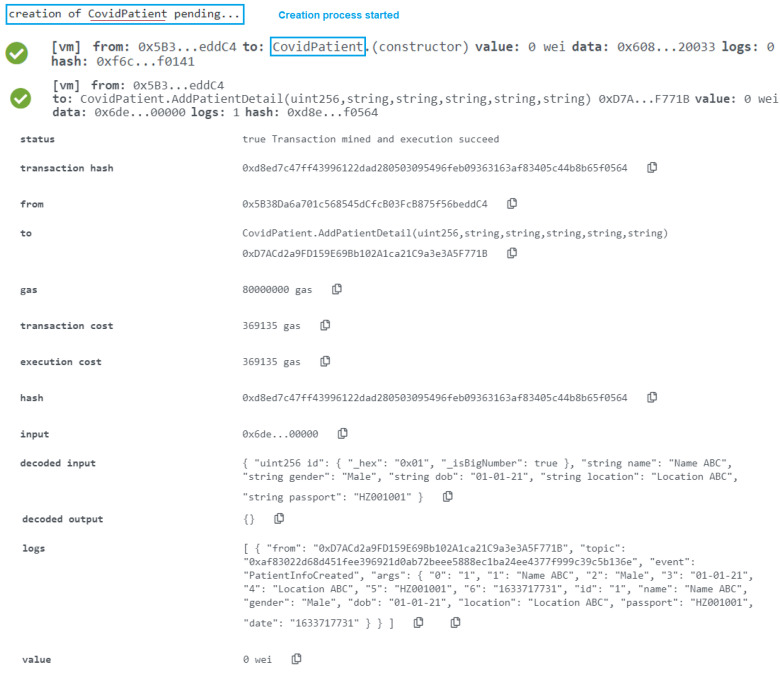
Save COVID-19 Patient’s detail in the blockchain ledger.

The user who is allowed in the CovidCenter can enter the details of patients who arrive for testing or immunization. As seen in Figure 10, the function was successfully completed, and the event now has the time in uint256. Any of the COVID-19 Testing/Vaccination Center’s patients can get updates. In order to make sure that the event generated as an announcement notice has all the necessary information, the function CovidPatientRecord was successfully assessed. The timing of the announcement, the information that must be broadcast, the patient’s smart contract address, the patient’s Ethereum address, and other data are shown in Figure 10.

*Add Covid Patient’s Detail*

**Figure 11:**
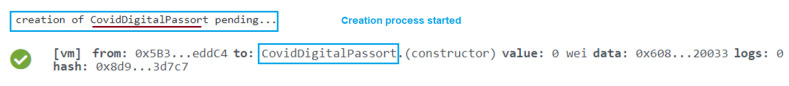

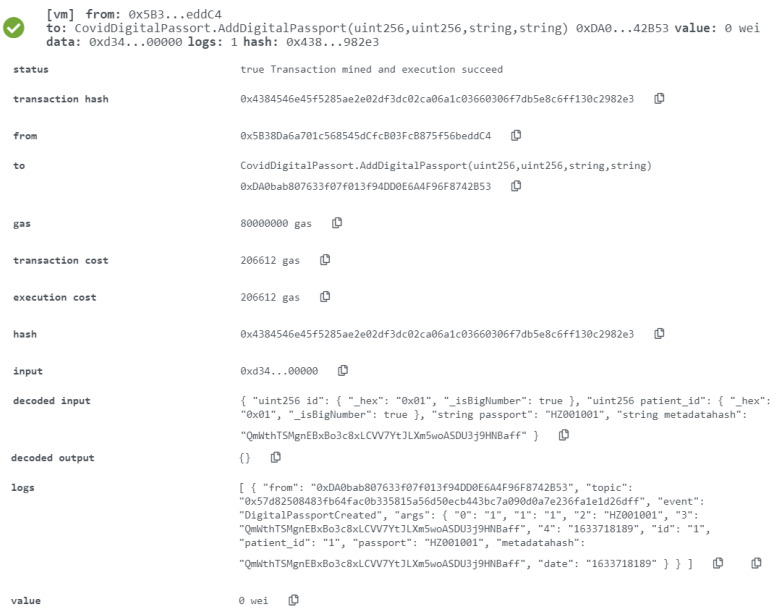
Upload COVID-19 DHP based on Testing/Vaccination and save detail in the blockchain ledger.

Following the collection of the COVID-19 lab test results, the function AddDigitalPassport was executed (see Figure 11). The event contains the Ethereum addresses of COVID-19 test and immunization participants, as well as the smart contract addresses, the IPFS hash of the test results, and the moment the event was published. The logs and event information are shown in the diagram above. When the digital health passport certificate is uploaded to IPFS storage, it generates a hash, which is then saved in a blockchain ledger with other patient information. Using the patient’s passport number, the patient can download a digital health passport from any location. The details of the patient can be verified immediately by the other linked and mentioned nations.

### 5.3. Add a Digital Passport on Decentralized Storage

**Figure 12:**
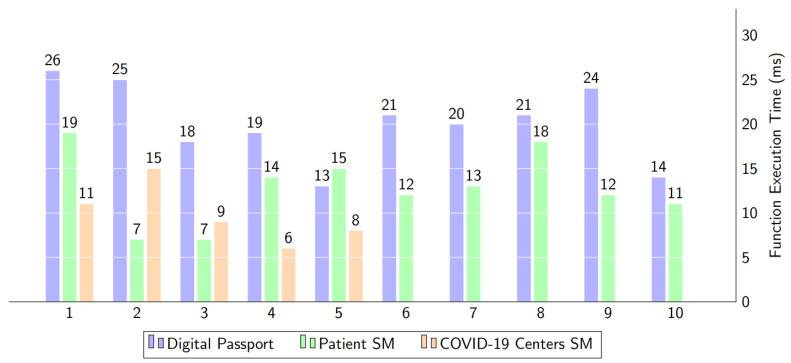
The computational time for data storage in IPFS and Blockchain via function execution.

The execution time of a smart contract is shown in Figure 12. Figure 12 displays the time required to download and upload the data from the IPFS and blockchain. The time spent on more than two smart contracts is shown on the digital passport bar. Along with the patient’s certificate hash and other crucial informational characteristics, the digital passport is uploaded into IPFS and subsequently preserved in the blockchain ledger. The test report for the patient’s smart contract is produced by the health center. This report displays the COVID-19 results, while the COVID-19 smart contract serves as a hub for the storage and validation of patient data.

**Figure 13:**
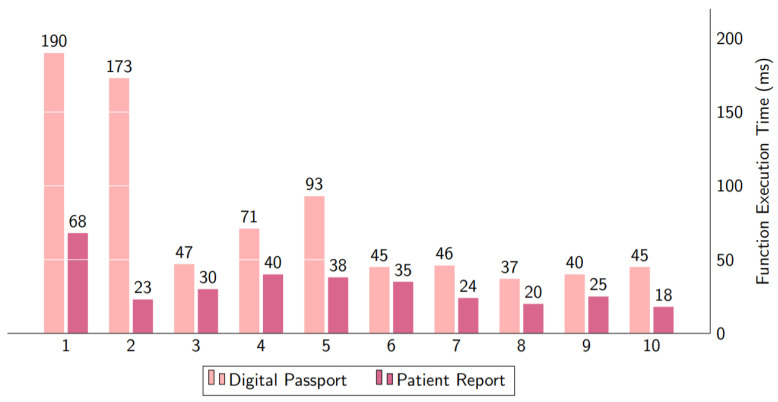
The computational time for accessing data from IPFS and Blockchain.

The amount of time needed to obtain data from the IPFS and blockchain is shown in Figure 13. The digital passport bar displays the time spent more than accessing the patient report. The digital passport is uploaded into IPFS and then saved into the blockchain ledger along with the patient’s file hash and other necessary information, while the patient report is merely kept in text form as a text on the blockchain ledger.

### 5.4. Evaluations of Query Response Time

Data searching is necessary for storing COVID-19 test and vaccination data to IPFS and maintaining record information in the blockchain. The query response time may be used to determine how well a system can store and retrieve data from a blockchain. We ran two tests; one to see how quickly the COVID-19 test and vaccination takers’ data were saved to the blockchain and the DHP certificate stored to IPFS, and another to see how quickly records with file hashes were saved to the blockchain. The result of query response time in milliseconds is shown in Figure 13, with the vertical axis indicating the response time in milliseconds and the horizontal axis representing the two execution functions. The “Complete function” displays the complete procedure, from recording tests and vaccine takers on IPFS, to saving record information to the blockchain using the medical data file hash. The delay caused by Metamask’s Smart Contract execution call is displayed in the “Smart Contract Function.”

**Figure 14:**
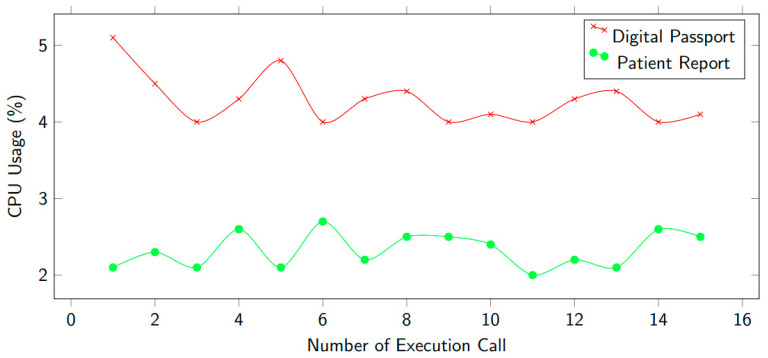
CPU consumption computation time.

The execution time for data access is shown in Figure 12 and Figure 14. The data is split into two categories. The first piece of data is the COVID-19 test and immunization certificate, which are obtained through a file hash from the IPFS. However, the execution time for numbers 1 and 2 was long since the execution is being started for the first time. The second part is to get the textual data of patients from the blockchain ledger.

In Figure 14, we depict the consumption of CPU utility while executing functions using smart contracts. There are various techniques including calling encryption data, storing in IPFS, and recording in the blockchain ledger. These tasks were completed in one cycle, after that we measured the CPU % usage. The decryption technique used the CPU much less. Figure 11 shows two scenarios: digital passport and patient report. It can be noticed that CPU consumption is higher for a digital passport.

Threats to Internal Validity: These refer to those constraints which influence the development and implementation of the suggested solution. For example, the number of sensors used to collect patient data. Decentralizing the blockchain-aided storage in IPFS, considering that the Ethereum framework using smart contracts for the evaluation of the proposed solution, can result in internal validity. It shows that changing the framework of Ethereum can cause different findings in the evaluation process. The research fraternity must consider a different framework than Ethereum which can substantially reduce the internal validity.

External Validity: This refers to the validity of a proposed solution on different linked systems and case studies. We used a case study-enabled technique for the validation of our solution. A single case study cannot be sufficient to realize the solution’s generality and consistent validity. In order to reduce the impacts of external validity, future studies must integrate other systems and more case studies.

## 6. Conclusions and Future Work

Decentralized designs and system interoperability have garnered a lot of attention as people have become more aware of the scattered nature of health care. The proposed system’s proof-of-concept architecture has been presented, which is a decentralized framework for storing and distributing COVID-19 Digital Health Passport Certificates based on Ethereum and IPFS. The performance of this framework has been assessed using a proof-of-concept based on the healthcare system. The system has been put into place on a permissioned Ethereum blockchain network that allows smart contracts.. In order to demonstrate the importance of the suggested model, the system’s performance has been contrasted with that of comparable models in terms of access control, scalability, and secrecy. This article discusses the development, implementation, and testing of a blockchain-based system for DHPs and immunity certificates. Infectious disease prevention in general and COVID-19 illness, in particular, are aided by the suggested technique. All of the pertinent smart contracts used in the paper have used on-chain storage as well as on-chain events and alerts. The participating entities’ individual Ethereum addresses in the system include self-sovereign identity, re-encryption, and pertinent data. We think that our approach lays the path for effective solutions that can aid in the prevention of infection transmission using tamper-proof tracking of accurate and timely events. A comprehensive cost analysis has been used to assess the algorithms for the suggested solution. The efficiency, logic, and practicality of the proposed method have been investigated and evaluated using an experimental implementation. While providing patients with a digital passport, the suggested approach also allows them access to an immutable medical database, resulting in increased efficiency, data provenance, and audit effectiveness. There are no third-party middlemen or administrative entities required since the data storage and exchange method are decentralized.

Limitation: Each transaction or transmission of data for uploading a health passport or immunity certificate is subject to an Ether cost because the proposed system depends on the Ethereum platform.

As a result, we will upgrade our decentralized solution with a multiple roles customization to allow multiple parties to safely and directly access the IPFS material via re-encryption techniques. The data will first be encrypted using a symmetric secret key by the data uploader. Only the hash of the encrypted data will be saved in the smart contracts, which will then be uploaded to IPFS. Patients can request to download their certificates through a secure channel, where the system generates two keys: private and public using a signature-based RSA algorithm. The private key will be kept by the patient, while the public key will be sent with a request and used by the admin (who gives a secret key access to the patient in order to decrypt the DHP certificate). The patient will then use their private key to decode the secret key and obtain the symmetric secret key. Finally, the patient will decrypt the material on IPFS using the symmetric key.

## Figures and Tables

**Table 1:** Identity management systems that are centralized and decentralized are compared.

Feature	Conventional	Blockchain
Governance	Centralized	Decentralized
Identity Change	Changing information stored on servers is a simple way to accomplish this.	It is possible to change one’s identity, but it is more difficult because it is within the user’s control.
Key Management	Resetting a password can be used to recover a password that has been lost.	The assets are lost if the private key is lost.
Storage	Centralized servers	Nodes are distributed.
Freedom	Users are at risk of having their identities stolen.	Users reclaim control over their personal information.
Date accuracy	Due to the asynchronous nature, it is possible to temporarily stale data.	Always up to date
Authentication	On behalf of users, the server takes action.	Cryptographic verification
Nature	Single point of failure	Distributed throughout nature
Data	No agreement is reached when new data is added via the administrator.	Only after an agreement has been obtained is fresh data uploaded.

**Table 2:** Cost analysis for SM.

Execution Type	Gas Used	Cost in Ether
SM Creation (CovidDigitalPassort)	556,046	0.01112092
SM Migration (CovidDigitalPassort)	27,363	0.0054726
SM Creation (CovidPatient)	1,787,213	0.03574426
SM Migration (CovidPatient)	27,363	0.0054726
SM Creation (CovidCenter)	856,103	0.01712206
Contract Migration (CovidCenter)	27,363	0.0054726
Initial Contract	225,237	0.0450474
Initial Migration Call	42,363	0.0084726
Final Cost		0.06849198

## Data Availability

Not applicable.
